# Supplementary material for: Serine Rejuvenated Degenerated Volvariella volvacea by Enhancing ROS Scavenging Ability and Mitochondrial Function
Source: J Fungi (Basel). 2024 Aug 1;10(8):540. doi: 10.3390/jof10080540 (PMC11355192; doi:10.3390/jof10080540)
Supplement: Supplementary file 1 [file jof-10-00540-s001.zip › supplementary materials.pdf]

Supplementary Material

1.1 Supplementary Figure

Figure S1. The tissue isolation process for succession strains is shown in corresponding images. (A) Original strains (T0), (B) Activation of strains, (C) Seed cultivation, (D) The original base, (E) Egg-shaped period, (F), Tissue separation of the fruiting body, (G) Tissue culture, (H) Strains preservation (T1-T19).

1.2 Supplementary Table

Table S1. Primers used for RT-qPCR

Gene	Symbol	Primer sequence（5’-3’）
Cu/Zn-sod	jgi|Volvo1|111092	ATGCGTTGCGCAACATTCGTCGCTG<br>AGCTGGTGTACGTCCAATGACACCA
Mn-sod1	jgi|Volvo1|114894	ATGGCCCACACTCTCCCTGA<br>TTAAAGCTTAGACTCGCCGG
Mn-sod2	jgi|Volvo|118151	CACAAAGACCGCTGCTATC<br>TAGTAACGACCTCTAGCTTGC
cat1	jgi|Volvo|113089	GCCGCATCGCCATTCTT<br>GCTTCACCCATACCCAACT
cat2	jgi|Volvo|116913	CCTTGCCCACTTTGACCG<br>TTGCCCTGACCTTCTTGC
gr	jgi|Volvo|113083	GCTGTCGTAGGTGCTGGGTA<br>GGGTCAAATCGCCTCAAA
gpx	jgi|Volvo|118375	TCGGAGGTGAATGGGAAC<br>TTGATCCTCGTCAGACCCATA
SPRYp	jgi|Volvo1|112937	CATTGCTTGTTCTACTGCC<br>ACCTTCAAACCCACCCTC
