# Supplementary figures and images for: Serine Rejuvenated Degenerated Volvariella volvacea by Enhancing ROS Scavenging Ability and Mitochondrial Function
Source: J Fungi (Basel). 2024 Aug 1;10(8):540. doi: 10.3390/jof10080540 (PMC11355192; doi:10.3390/jof10080540)

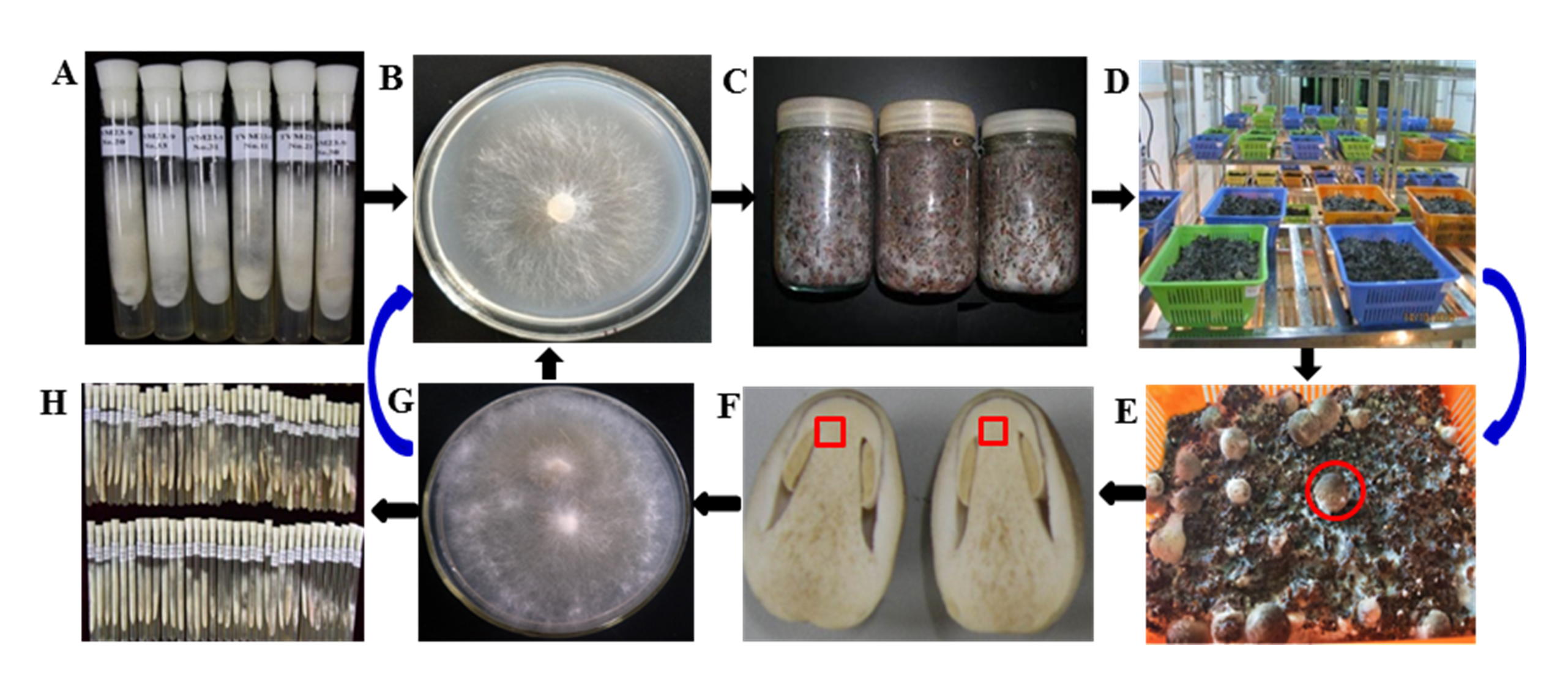

Supplement: Supplementary file 1 [file jof-10-00540-s001.zip › Supplementary Figure S1.tif]
